# Self-produced signs in decision-making and contextual correlations

**DOI:** 10.3389/fpsyg.2026.1838592

**Published:** 2026-07-09

**Authors:** Leopoldo Trieste, Giuseppe Turchetti

**Affiliations:** Institute of Management, Sant'Anna School of Advanced Studies, Pisa, Italy

**Keywords:** choice, contextuality, decision-making, quantum correlations, signs

## Abstract

We challenge traditional decision theories centered on external stimuli by adopting a historical-cultural perspective that distinguishes between received stimuli and self-produced signs. Our focus is on decision contexts in which self-produced signs are actively generated to support cognition, reflecting its constructive and goal-directed nature. We investigate whether combinations of these signs can give rise to non-classical correlations without invoking artificial superpositions of presented options. This perspective departs from classical, preference-centric models that interpret decisions as the resolution of fixed and pre-existing latent preferences. Instead, we propose a contextual, non-preference-based framework in which choices emerge from the dynamic generation of correlations among self-produced signs. In this view, self-produced signs actively constitute the decision context itself. While outcomes remain locally interpretable within a classical perspective, interactions among locally consistent processes can generate global incoherence. Such incoherence is not accessible at the level of individual cognition. This approach reframes decision-making as an active, context-driven process shaped by the interplay between self-generated signs and situational constraints. By shifting the focus from static preferences to context construction, it provides a novel framework for understanding complex and non-classical patterns in human decision-making beyond traditional models.

## 1 Introduction

The current literature on decision-making and choice mainly focuses on how environmental stimuli and stimuli directly originating from the objects of choice influence decision-making. We address the limitations of this passive role of decision-maker by following the historical-cultural approach that distinguishes between received stimuli and self-produced signs, which aid decision-makers in making choices and differentiate human beings from other animals.

This approach allows a more natural foundation of contextual correlations in decision-making. We examine decision-making contexts in which self-produced stimuli rely both on the objective properties of the alternatives and on measurements of the decision-maker's behavior. We investigate whether and under which conditions combinations of self-produced stimuli can generate contextual (i.e., non-classical probabilities), and quantum-like correlations, evidenced by violations of Bell's inequalities.

The reason for contextual correlations via self-produced stimuli appears more natural than the current quantum-decision making that seems a technique forced to be applied in decision-making.

## 2 Different approaches to quantum physics and quantum decision theory

### 2.1 Perspectives in quantum physics

In the physical literature, several different approaches make use of the quantum formalism, but they differ substantially in their interpretative commitments (Norsen, 2017).

A first broad class of approaches, inspired by the Copenhagen interpretation and Bohr's operational stance, treats quantum theory primarily as a predictive framework. Rather than describing an underlying reality, the theory is taken to assign probabilities to possible outcomes within a given measurement context. Central to this view are contextuality and the inseparability of system and measurement, while questions of ontology are deliberately set aside.

At the opposite end of the spectrum lies the Bohmian approach, which is explicitly ontological and deterministic (Bohm, 1952). In Bohmian mechanics, hidden variables are located in the state of the system itself, and quantum effects arise from a non-local dynamic governed by the quantum potential. While conceptually clear, this approach assumes a strong underlying physical ontology and is therefore rarely adopted in cognitive or decision-theoretic contexts.

On this line quantum physics is not intrinsically probabilistic but fundamentally deterministic at a deeper level. The apparent indeterminacy of measurement outcomes does not reflect true ontological randomness, but rather limitations in the standard formalism.

Observers' choices in experiments (what to measure) are not truly free in an absolute sense; any choice is entangled with the state of the universe.

In other words, if an observer were to make a different measurement choice, the entire state of the universe would have to be different, meaning that free will is strongly constrained by the deterministic underlying reality.

Thus, what we perceive as “free choice” is limited to relative decisions within the constraints of the universe's deterministic evolution, not absolute autonomy ('t Hooft, 2007).

The approach developed by Aerts (1992, 1998), which is adopted in this paper, occupies a distinct intermediate position. It is explicitly realist, yet fundamentally contextual. Within the Hidden Measurement Approach, quantum probabilities do not arise from ignorance about the system's state, but from an objective indeterminacy inherent in the measurement interaction itself. The hidden variables are not properties of the system, but of the measurement context, representing uncontrollable fluctuations in the actualization of the interaction between system and context in each measurement.

This perspective preserves realism while abandoning determinism, and provides a natural account of interference, contextuality, entanglement, and violations of Bell inequalities without postulating non-local hidden variables in the system's intrinsic dynamics. Measurement does not reveal a pre-existing property in isolation; rather, pre-existing properties of the system, together with the contextual features of the measurement interaction, jointly generate the observed quantum correlations.

However, when considering extensions to macroscopic domains, the presence of shared operational resources or experiments that are mutually exclusive among different contexts may introduce effective violations of the no-signaling condition. In such cases, contexts can become operationally coupled, allowing partial accessibility to global structure and thereby limiting the emergence of genuine quantum correlations. These signaling effects do not arise from fundamental non-local interactions, but from contextual dependencies induced by resource constraints, which may restrict the applicability of a fully quantum interpretation at the macroscopic level.

### 2.2 Quantum decision theory

Quantum Decision Making (QDM) (Busemeyer and Bruza, 2012) is an interdisciplinary framework that uses the mathematical formalism of quantum theory to model cognitive processes. It does not assume physically quantum minds or hidden variables but adopts quantum tools instrumentally to capture contextual effects and deviations from classical probability.

Unlike classical decision theory, QDM relies on a non-Boolean probabilistic structure. Mental states are represented as vectors in a Hilbert space, encoding potentialities rather than definite preferences. Decisions are modeled as measurements that actualize one of these potential outcomes, implying that preferences are not pre-existent but context dependent.

A key feature is contextuality: outcome probabilities depend on the decision context, including order and framing effects. This leads to non-commutativity of decisions, meaning that answering A then B may differ from answering B then A. Consequently, no single joint probability distribution exists for all decisions.

In multi-agent or multi-attribute settings, QDM uses tensor-product structures, allowing for entanglement. Entangled states represent holistic dependencies that cannot be reduced to independent components, explaining phenomena such as violations of the sure-thing principle and complex interdependencies beyond classical models.

QDM adopts a flexible notion of rationality, allowing violations of classical axioms while maintaining internal consistency within a quantum probabilistic framework. Observed correlations are not due to hidden variables but reflect intrinsic contextual structure.

### 2.3 Limitations

A key limitation of QDM is its preference-centric nature: it models decision-making as a superposition of preferences, with choice acting as a collapse process. However, this remains largely formal. The framework explains contextuality through mathematical structure but does not always provide a clear cognitive or mechanistic account of its origin. QDM is often seen as a descriptive rather than fully explanatory theory. Additionally, it can reinforce a passive view of the agent: decisions appear as measurements of pre-existing superpositions, leaving limited room for agency, control, or creative construction of preferences.

## 3 Non-classicity and quantum correlations

The introduction of QDM induces reflection on the way, manner, and conditions under which non-classical correlations emerge and how they can be detected. It is important to distinguish among different notions of non-classicality: Bell non-locality (Bell, 1964), Wigner-type quasi-probabilistic approaches (Wigner, 1932; Kenfack and Życzkowski, 2004), and Kochen–Specker contextuality (Kochen and Specker, 2011).

### 3.1 Violation of Bell's inequalities

In the Bell's approach, the hierarchy is as follows: (1) one observes a set of correlations between measurement outcomes; (2) one then asks whether these correlations can be explained by a local hidden-variable model, i.e., in terms of underlying local correlations.

We adopt the CHSH version (Clauser et al., 1969) of Bell's inequalities.

Let consider two subjects A, and B, that perform two types of experiments: e1 e2 by subject A; e3 e4 by subject B. e1 and e3, e2 and e4 are the same experiments, respectively, made locally by the two subjects. Each test produces two discrete outcomes: positive or negative.

A correlation function among the couples of experiments, one from A and one from B *x, y*, is defined as:


$$\begin{array}{l} E\left(xy\right)=p_{++}\left(xy\right)+p_{--}\left(xy\right)-\left(p_{+-}\left(xy\right)+p_{-+}\left(xy\right)\right) \end{array}$$

as the sum of probabilities that *x* and *y* are coherent (i.e., when *x* = +, *y* = + and when *x* = −-, *y* = −), minus the probability of observing opposite outcomes).

Let's also consider the quantity:


$$\begin{array}{l} Q=E\left(e1e4\right)+E\left(e2e3\right)+E\left(e2e4\right)-E\left(e1e3\right) \end{array}$$

If |*Q*| ≤ 2, the correlation of observations depends on a past cause (hidden variable). Otherwise, there is a non-classical correlation among observations that is not generated by a past common cause but by the experiment itself (Aerts et al., 2000).

A direct and computational way to detect CHSH is considering matrix *M* of correlations and adopting the Horodecki's approach (Horodecki et al., 1995). $Q_{\max\;}=2\sqrt{u_{1}+u_{2}}$ where *u*_1_, *u*_2_ are the largest eigenvalues of the matrix *M*^*T*^*M*.

In the framework of Bell inequality violations, entanglement can be understood as the presence of correlations between observed outcomes that cannot be explained by independent, locally defined properties. In the CHSH formulation, the central question is whether such correlations admit a description in terms of local hidden variables, i.e., variables predefined independently at each measurement setting. A violation of this assumption implies that no such classical description is possible.

Observing a violation of Bell inequalities, i.e., |Q| > 2, requires that the different measurement conditions cannot be treated as jointly realizable, since performing one test may affect the outcome (or even the definition) of others. However, this condition alone is not sufficient. A violation occurs only when the observed correlations satisfy specific quantitative constraints and are sufficiently strong to exceed the classical bound.

#### 3.1.1 Quantum and super quantum non-classical correlations

A violation of the CHSH inequality has physical quantum-mechanical interpretation only when $2<|Q|\leq 2\sqrt{2}$ (the Tsirelson's limit). In this range, the observed correlations are non-local and can be realized by a quantum state. When ∣*Q*∣ = 4, correlations are still non-local and satisfy the no-signaling principle, but they cannot be produced by any quantum state. Such correlations are therefore called post-quantum (or super-quantum) correlations.

#### 3.1.2 The no-signaling condition

All the above conditions assume that there is no signaling between contexts. In a bipartite scenario with two separated systems, Alice (A) and Bob (B), described by conditional probabilities *P*(*a, b*∣*x, y*), the no-signaling condition requires that each party's local statistics are independent of the other party's choice of measurement, i.e., *P*(*a*∣*x, y*) = *P*(*a*∣*x*) and *P*(*b*∣*x, y*) = *P*(*b*∣*y*). A violation of no-signaling occurs if Alice's marginal distribution depends on Bob's setting (or vice versa), meaning that *P*(*a*∣*x, y*)≠*P*(*a*∣*x*), which would imply the possibility of communication through measurement choices. When a single subject performs the analysis of two separated systems, the no-signaling condition still applies as a constraint on the operational probabilities, provided the systems remain physically independent; the identity of the observer is irrelevant, since what matters is the locality of the measurement operations on distinct subsystems. If this operational independence is satisfied, the model can exhibit violations of Bell inequalities (such as CHSH > 2), showing non-classical correlations that cannot be explained by local hidden variables, while still respecting the no-signaling condition. Conversely, if no-signaling is violated, Bell-type analysis loses its standard physical meaning, since the observed correlations can be reproduced by direct communication between the subsystems rather than genuine non-locality (see Table 1).

**Table 1:** Types of models, CHSH constraints (with two agents), and the no-signaling condition.

Model	CHSH	No-signaling
Local classical	Q ≤ 2	Yes
Quantum	2 < Q ≤ 2√2	Yes
PR-box (post-quantum)	4	Yes
Signaling models	up to 4 (not bounded by Bell constraints)	No

### 3.2 Pseudo-probabilities

Let's consider two contexts *C*_1_, *C*_2_ in which some experiments produce outcomes (*x*_1_, …*x*_*n*_|*C*_1_), (*x*_1_, …*x*_*n*_|*C*_2_), respectively. If the probability distribution within a context, *P*_*C*_(*x*_1_, …*x*_*n*_|*C*_1_) is normalized to 1 and some probabilities *P*_*C*_(*x*_*i*_|*C*_1_) are negative, then we are in the regime of local contextuality (sometime called quasi-quantum correlations. We do not consider this case here. Our focus is on whether a global joint distribution *P*(*x*_1_, …, *x*_*n*_) among contexts (within contexts probability is classical) exists capable of reproducing all contextual marginals.

If there exists a global probability distribution P(*x*_1_, …*x*_*n*_) such that P(*x*_*i*_)≥0, for each *x*_*i*_ and $\sum_{i=1}^{\;n}p\left(x_{i}\right)=1,\;$capable of reproducing all marginals, then we are considering a classical probability regime.

In all the other cases we are in a condition of contextuality; that is when (*P*(*x*_1_, …*x*_*n*_) must describe all observed outcomes, a global normalized $\sum_{i=1}^{\;n}P\left(x_{i}\right)=1\;$require that some probabilities must be negative (Fine, 1982).

#### 3.2.1 Interpretation of negative probabilities

A useful Bayesian intuition for negative probabilities (see, among others, Trieste and Turchetti, 2024) is in terms of betting: a negative probability corresponds to an outcome you are so confident will not occur that you would require payment to participate in a bet on it. For example, if *P*(*E*) = −0.2, you would need to be paid 0.2 units to bet on the event *E*, because the system is over-constrained and waiting for *E* would be a guaranteed loss.

In this way, negative probabilities are not non-sensical numbers; they encode the degree of contextual over-determination: certain outcomes cannot consistently exist across all measurement contexts. Of course, we must modify Kolmogorov axioms of probability as a non-negative measure.

All in all, it is better to keep negative probabilities than to transform them into positive values.

If we adopt distributions that are not linear in probability to avoid non-negative probabilities, then because the map is non-linear, the sum over all outcomes may not equal 1 (e.g., violation of standardization).

Maintaining linearity and negative probabilities keeps the global distribution as a linear combination of deterministic assignments (convex-linear model) where all marginals and correlators are linear functions of the hidden-variable weights, negative probabilities appear when classical positivity is impossible (either in marginals or in the full joint distribution), the interpretation is transparent: negative numbers correspond directly to the impossibility of a classical explanation and this preserves the convex-linear structure.

Trying to remove negativity by using a non-linear mapping to force probabilities to be positive does not allow the linear correspondence between hidden-variable weights and observables; the global probability may sum to more than 1 or less than 1, so it ceases to be a proper probability distribution; marginals and correlators become non-linear functions of the hidden variables, making interpretation more obscure. Also, contextuality is hidden, i.e., it becomes less transparent, but it cannot be removed.

The Farkas' lemma (Stoer and Witzgall, 1970) provides necessary and sufficient conditions for a linear system of the form *Ax* = *b* to admit a solution with all components non-negative, i.e., *x*_*i*_≥0. The lemma states that either the system *Ax* = *b* admits a solution with all components non-negative, or there exists a vector *y* such that *A*^*T*^*y*≥0 and *b*^*T*^*y* < 0, but never both.

In this pseudo-probability approach, one starts from locally defined probability distributions and then seeks a global distribution capable of reproducing these local marginals. When such a global distribution cannot be constructed without introducing negative values, the underlying behavior departs from classicality.

### 3.3 Kochen–Specker (KS) contextuality

Kochen–Specker (KS) contextuality formalizes a fundamental limitation on assigning values to quantum observables. The key idea is the following: one can consistently assign definite values to observables only within a single measurement context, that is, within a set of mutually compatible (jointly measurable) observables. However, when one attempts to extend these assignments across different contexts, requiring that each observable has a fixed value independent of the context in which it is measured, contradictions inevitably arise. The logic behind this result is that different contexts impose compatibility constraints that cannot all be satisfied simultaneously by a single global assignment. In other words, each context can be thought of as locally consistent, but these local assignments cannot be combined into a single, context-independent description. This shows that measurement outcomes cannot be understood as revealing pre-existing properties that are independent of the measurement setting. Instead, the value attributed to an observable necessarily depends on the context in which it is measured.

### 3.4 Relationship among different approaches

The various notions of non-classicality (Bell, quasi-probability representations, Kochen–Specker contextuality) can be understood as different manifestations of the same underlying obstruction to a global classical description. In the KS framework, this obstruction appears at the logical level: it is impossible to assign definite, non-contextual values to all observables in a way that is consistent across measurement contexts. When this problem is reformulated in probabilistic terms, it translates into the non-existence of a global joint probability distribution that remains positive; if such a representation is nevertheless enforced, it must involve negative probabilities, as in Wigner-type approaches. Finally, this failure can be expressed operationally through linear constraints on observable correlations, leading to Bell or contextuality inequalities: their violation provides an experimentally testable signature of the same underlying non-classical structure.

## 4 Types of stimuli and choice: some preliminary definitions

After these preliminaries we can start to describe our non-preference centric approach to decision-making.

This approach assumes a positive and creative role of decision-making, the central role of measures to generate contextuality, no superposition of alternatives of choices but properties that are objectively satisfied by the different contexts generated by measures.

These properties and the types of measures generate non-classical i.e., contextual correlations. In this the perspective is in line with the Hidden Measurement Approach (Aerts, 1998; Aerts et al., 2000).

Our perspective is a measure-centric approach where decision-makers are free and active in selecting or inventing experiments. Measures are not choices but means to make choice.

### 4.1 The historical-cultural paradigm

In decision-making models, agents process stimuli from external sources (e.g., other agents, the environment). While behaviorism (Watson, 1913; Skinner, 1965), cognitivism (Gardner, 1987), and other perspectives (Hilgard, 1987; Hillner, 1984) only in part recognize the role of decision-makers in processing stimuli assuming a sort of *passive determinism*, the phenomenological and the historical-cultural school emphasize the active role of the decision-maker (Kozulin, 1984; Wertsch, 1985). Gestalt psychology also highlighted humans' active role in perceiving stimuli and solving problems (Wertheimer, 1959).

The historical-cultural paradigm (Vygotsky, 1977, 1979) observed that humans systematically produce self-stimuli during decision-making. While existing literature focuses on how stimuli and specific conditions such as scarcity enhance creativity (Mehta and Zhu, 2016; Holbrook and Hirschman, 1982), we emphasize the systematic use of creativity in decision-making through the production of self-produced sign as artificial triggers of choice. We show how the system of adopted signs may generate correlations that cannot be interpreted classically.

### 4.2 Types of stimuli and the nature of objects of choice

Literature classifies stimuli as external, categorizing them by sensory systems (visual, auditory, olfactory, taste, tactile) processed through neurons and cortical areas (Kandel et al., 2012). The neuroscience perspective (Karmarkar and Plassmann, 2019) focuses on quantitative comparisons but misses qualitative difference. Other classifications consider stimuli's role in decision-making, distinguishing between environmental and choice-related inputs, or by source, physical/natural vs. cultural/historical.

From our view, a product is an “open work,” an artwork intentionally (or unintentionally) designed to require active user participation (Bianchi, 2006; Cova and Dalli, 2009; Eco, 1989).

### 4.3 Given stimuli

From the decision-maker's perspective, given stimuli are elements of the environment or stimuli produced by other agents. We do not explore this type of stimulus in depth. Given or neutral stimuli are the least intriguing dimensions for decision-makers. In fact, when processing given stimuli, the qualitative difference between human beings and other animals diminishes.

### 4.4 Signs

Signs are self-produced stimuli shaped by social and cultural environments, guiding judgment, problem-solving, and decision-making (Vygotsky, 1977). They reduce uncertainty and clarify the relationship between an object and its meaning. Signs also result from experiments suggesting decisions, incorporating elements of the product, environment, or agent.

According to Mick (1986) and Hawkes (1977), self-produced signs are classified as indexes (linked to objects), icons (similar to objects), and conventions (arbitrary). Note that from this approach, heuristics and utility maximization are both signs i.e., self-produced stimuli to make choice. Signs can be interpreted also as *interrogative acts*.

### 4.5 Choice

We define choice as the process through which a decision-maker selects among different alternatives. A decision-making process can be seen as the application of signs as real or mental experiments. These signs act as shortcuts in the interpretative process necessary for making a choice.

## 5 An operational context-dependent structure

The present approach is naturally framed within the quasi-probability formalism: starting from locally consistent distributions, one seeks a global representation capable of reproducing them, which, however, can only be achieved at the price of introducing negative probabilities. In this sense, it can be understood as a probabilistic reformulation of Kochen–Specker contextuality. At the same time, it is consistent with a contextuality-by-default perspective (Dzhafarov et al., 2015; Dzhafarov and Kujala, 2016), in which measurements performed in different contexts are treated as fundamentally distinct random variables, and contextuality emerges from the impossibility of constructing a globally consistent coupling across these contexts.

### 5.1 Primitive objects: acts, not things

In the proposed framework, there are no observations as passive entities. The primitive elements are interrogative acts or experiments *e*_1_, …*e*_*n*_ which generate self-produced signs, and contexts *C*_*i*_ which arise from the composition of such acts. An act is an operation on the traces of previous interrogations. These traces are not treated as static objects, but as operational residues that structure the space of possible future responses.

A minimum context is defined as the composition of two acts.


$$\begin{array}{l} C_{ij}=e_{i}{}^{\circ}e_{j} \end{array}$$

### 5.2 Non-commutability

Composition is non-commutative:


$$\begin{array}{l} e_{i}{}^{\circ}e_{j}\neq e_{j}{}^{\circ}e_{j} \end{array}$$

This non-commutativity is not an additional assumption but follows from the fact that each act transforms the conditions under which subsequent acts operate.

Each composed context produces a sign. Because of bounded rationality, the outcome is binary, or it can be translated into a binary response.

No deeper ontological structure is assumed beneath these outcomes. They are irreducible local results of context-dependent compositions of acts.

If an act *e*_*i*_ appears in multiple contexts, it must maintain operational coherence. The same act cannot yield incompatible statistical behavior across different compositions. However, its effect is permitted to vary depending on the surrounding context. Thus, each act is stable as an operation but context-dependent in its realized effects. This is a global principle of consistency under shared compositions: an act cannot behave arbitrarily differently across contexts; yet its realized behavior is modulated by the structure of the context in which it appears. This constraint replaces classical notions of state consistency.

This framework describes how interrogative acts compose, which compositions are admissible, and which global structures emerge from overlapping local compositions.

Correlations among signs are not fundamental; they emerge from the statistical structure of composed acts. Thus, correlation is an emergent property of a non-commutative operational structure, rather than a primitive relation between pre-existing variables.

#### 5.2.1 Emerged geometry

From these assumptions, a geometric structure becomes unavoidable. Acts cannot be represented as independent entities. Composition induces relational constraints, and overlapping contexts generate a structured space of dependencies. The minimal structure satisfying non-commutativity, local coherence, binary saturation, and symmetry constraints, induces an implicit geometric structure analogous to the angular constraints found in quantum correlations.

### 5.2 Separation, false separation, and no signaling

A crucial consequence of the non-commutative structure is the possibility of false separation. A separation between *A* and *B* is false when it appears stable within a restricted set of contexts, but fails under different compositions of acts, revealing hidden interdependencies. Formally, a partition is false if its stability is not preserved under recompositing.

This implies that separability is not absolute, independence is context-dependent, and classical structure may emerge only locally. In this framework, the no-signaling principle does not eliminate global structure. Rather, it ensures that such structure cannot be used to controllably influence local probability distributions.

### 5.3 Relevant implications

The theory eliminates classical ontological assumptions: no underlying world state, no fixed observables, no passive measurements. Instead, reality is defined as a network of interrogative acts whose compositions generate locally consistent outcomes constrained by global coherence. The most radical implication is that reality is not what exists independently of inquiry, but the structure of all possible coherent compositions of interrogative acts. Thus, probability does not describe a state of the world, but the operational compatibility among composed acts. Even the notion of separation is not primitive. To assert separation between *A* and *B* is itself an act. Separation is therefore not a pre-existing property, but the result of an operation that constructs a partition of possibilities.

### 5.4 Provoking nature or provoking our beliefs

Human beings believe that when we provoke nature, we conduct experiments to uncover its mysteries. Yet the outcomes of these experiments reveal only a small part of what remains inaccessible.

An act does not transmit information about a pre-existing state. Instead, it reveals the structure of the context through the transformations it induces on subsequent acts.

A question functions as a sign because it operates on the very system that generates it, modifying the contextual structure and leaving traces that reappear in future interrogations.

Every interrogation and experiment reveals how previous acts give rise to new ones.

We may call “nature” a relatively stable system of self-produced signs that, from our perspective, appears not to modify the local framework of questions and signs. Nature is the ensemble of our active operations.

Within this framework, computing ∣*Q*∣ is itself an interrogative act. It introduces a mode of aggregation over outcomes and reshapes the space of admissible compositions.

Interrogative acts do not belong to fixed categories:

- an act is structural if it modifies the algebra of compositions
- an act is internal if it operates within a stabilized algebra

This distinction is context-dependent and emerges from non-commutative compositions.

Finally, identifying non-classicality is itself an interrogative act. It introduces criteria for comparing compositions and thereby helps define the very conditions under which quantum behavior becomes meaningful.

### 5.5 No-signaling

From a local perspective, the subject does not experience their own interrogative acts as operations that shape outcomes. Instead, outcomes are perceived as properties of the object. This illusion arises because local statistical distributions are independent of distant contextual choices, making the transformative role of acts operationally invisible.

The no-signaling condition ensures that local outcome distributions remain independent of distant interrogative choices. As a consequence, the subject has no operational means to detect the influence of such choices. This gives rise to an apparent objectivity: outcomes are naturally interpreted as intrinsic properties of the observed objects rather than as effects of context-dependent interrogations. In this sense, no-signaling corresponds to the local invisibility of the impact of acts, or the neutrality of investigation. *No-signaling is the operational invisibility of contextual interventions at the local level*. Therefore, the no-signaling principle guarantees the perceived neutrality of acts, but not their structural neutrality.

In classical theories, correlations are explained by a shared underlying cause. In contrast, in the present approach, no common cause is assumed. Instead, global structure may emerge from the composition of local acts, without being reducible to any underlying variable, and remains inaccessible at the level of local observations.

### 5.6 Time and composition of acts

Time emerges from the ordered composition of acts. Non-commutativity makes this ordering physically meaningful, as different sequences of acts produce different contexts.

In this framework, time is not a primitive parameter, but the ordering structure induced by the composition of acts. Temporal reversibility can be understood as a property of the compositional structure of acts. If there exist sequences of interrogative acts whose composition restores the initial context, up to operational equivalence, then the system admits a form of reversibility.

More precisely, if distinct compositions lead back to a context that is indistinguishable with respect to subsequent interrogations, then the order of acts does not introduce an irreversible loss of structure. In this sense, time does not appear as a primitive parameter, but as an emergent ordering of acts whose reversibility depends on the existence of restoring compositions.

This provides an operational analog to relativistic reversibility: just as physical laws in relativity are invariant under time reversal, here the structure of acts remains invariant under compositions that effectively invert prior contextual transformations.

Conversely, when no such restoring composition exists, prior contexts cannot be reconstructed. This loss of reconstructability induces an arrow of time, not as an external feature, but as a structural asymmetry in the space of act compositions.

### 5.7 A multi-agent setting

In a multi-agent setting, the status of interrogative acts changes fundamentally. A subject does not have direct access to the acts performed by others, but only to the signs that result from those acts. These signs enter the observer's own operational context and become part of subsequent compositions. In this sense, other subjects do not appear as sources of acts in the same way as oneself, but as sources of externally generated signs whose internal generative structure remains inaccessible.

This asymmetry implies that interaction cannot be described as a simple composition of independently accessible operations. When a subject performs an act in the presence of another agent, the resulting sign reflects not only the local interrogation, but also the influence of acts performed by the other. However, this influence cannot be uniquely decomposed: the contribution of each agent is not operationally separable. What is observed is a composite outcome embedded in a shared context, rather than a sum of independently identifiable effects.

The introduction of multiple, operationally isolated subjects has significant consequences for the structure of correlations. In such a setting, each subject has access only to the signs produced by others, not to the interrogative acts that generate them. This restriction fundamentally alters the nature of compositional structure: contexts are no longer formed solely by the composition of locally accessible acts, but by the integration of externally generated signs whose internal origin remains hidden. Consequently, correlations cannot be understood as relations between jointly accessible operations. Instead, they arise between locally performed acts and externally received signs that cannot be decomposed into independent contributions. This introduces a deeper form of non-separability, not only between acts but also between agents. The impossibility of reconstructing how a given sign was generated implies that no global description can assign consistent roles to each subject's contribution.

### 5.8 Interpretation shapes reality: local outcomes and global structure

The interpretation of local outcomes determines the structure of the theory. If observed outcomes are treated as signals revealing an underlying global state, then a classical description follows, based on pre-existing properties and a consistent global assignment. However, if local outcomes are instead interpreted as context-dependent and not as indicators of any underlying global structure, then no global description can be constructed. In this case, correlations arise from the relational structure between contexts, and quantum-like behavior naturally emerges. Thus, the distinction between classical and quantum descriptions does not lie in the observed data, but in whether local outcomes are taken to carry information about a global state or are understood as intrinsically contextual. If one restricts attention to individual local contexts, all observed statistics admit a classical description. Quantum-like behavior arises only when one attempts to combine multiple local contexts into a single global representation. It is precisely the impossibility of constructing such a global description that gives rise to non-classical correlations.

#### 5.8.1 A unified description of correlations

We introduce a unified description of correlations based on two fundamental parameters: local accessibility A and global coherence C, both defined in the interval [0,1]. Accessibility A measures the extent to which the global structure is reflected in local observations, while coherence C quantifies the degree to which a consistent global structure exists. From these quantities, we define a single normalized parameter.


$$\begin{array}{l} \Delta =\left(1-A\right)\left(1-C\right) \end{array}$$ (1)

which represents the fraction of global inconsistency (non-classicity) that remains locally inaccessible.

Let's $\omega :\;=\;\sum_{\text{λ}}:\;w\left(\text{λ}<0\right)|w(\text{λ})|$ and $C:\;=\;\frac{1}{1+\omega }.$ The quantity *W*(λ) represents a global quasi-probability distribution defined over a set of hidden configurations λ. Each λ corresponds to a complete assignment of outcomes to all observables under consideration.

The quantity *A*∈[0, 1] measure the *operational access* to the underlying global structure. We define *A*in terms of signaling as


$$A:\;=\;\underset{X,\;c,c^{\prime }}{\max}\frac{1}{2}\sum_{x}|P(X=x|c)-P(X=x|c^{\prime }|\;,$$ (2)

which measures the maximal variation of the marginal distribution of an observable *X* under different measurement choices *c* and *c*′ made by other agents. In Equation 2, A quantifies the degree of signaling, i.e., the extent to which local outcomes depend on external context choices. In particular, *A* = 0 corresponds to the no-signaling regime, in which all contexts yield identical marginals and are therefore operationally indistinguishable, although still conceptually distinct.

Accordingly, 1−*A* measures the degree of no-signaling, that is, the extent to which the global structure remains hidden from local observations.

The strength of correlations can then be constrained in terms of Δ through the bound


$$\begin{array}{l} Q\leq 2\sqrt{1+\Delta^{2}} \end{array}$$ (3)

which naturally reproduces the classical and quantum regimes. When Δ = 0, either because the system is globally coherent (C = 1) or fully accessible (A = 1), the bound defined in Equation 3 reduces to Q ≤ 2, corresponding to classical correlations. As Δ increases, hidden incompatibility grows, allowing stronger correlations. The maximal quantum value Q = 2√2 is reached when Δ = 1, corresponding to maximal global inconsistency that remains completely inaccessible locally.

This formulation shows that quantum correlations emerge from a precise interplay between global structure and local limitations. The key is not merely the presence of inconsistency, but its inaccessibility: only incoherence that cannot be locally resolved contributes to non-classical effects. In this sense, Δ provides a natural coordinate for the space of correlations, and the quantum bound appears as the maximal value compatible with a geometric constraint on how hidden inconsistencies can combine.

## 6 Self-produced signs and contextual correlations

### 6.1 Multi agents

Consider two independent observers, Alice and Bob, evaluating the same consumer product under a set of well-defined criteria. Each observer has access to two interrogative acts, applied consistently across trials. For instance, Alice may assess whether a beverage produces a rapid energy boost (A) or has an innovative taste (A′), while Bob evaluates whether the effect is stable over time (B) or whether the drink is immediately pleasant (B′). These criteria prove distinct aspects of the same product. While each question yields stable local statistics, their combinations reveal correlations that cannot be reconciled with a single set of intrinsic properties.

Each interrogative act or self-produced sign is locally identical across contexts: whenever Alice applies A, she does so under the same conditions, independently of which act Bob performs. The same holds for Bob. As a result, local distributions remain stable. In particular, the probability that Alice assigns a positive outcome under A is the same regardless of whether Bob applies B or B′. Formally, this corresponds to the no-signaling condition:


$$\begin{array}{l} P\left(a|A,B\right)=P\left(a|A,B^{\prime }\right) \end{array}$$ (4)

and similarly for Bob. From the perspective of each observer, the evaluation appears objective and independent of external influences (see Equation 4).

**Figure 1:**
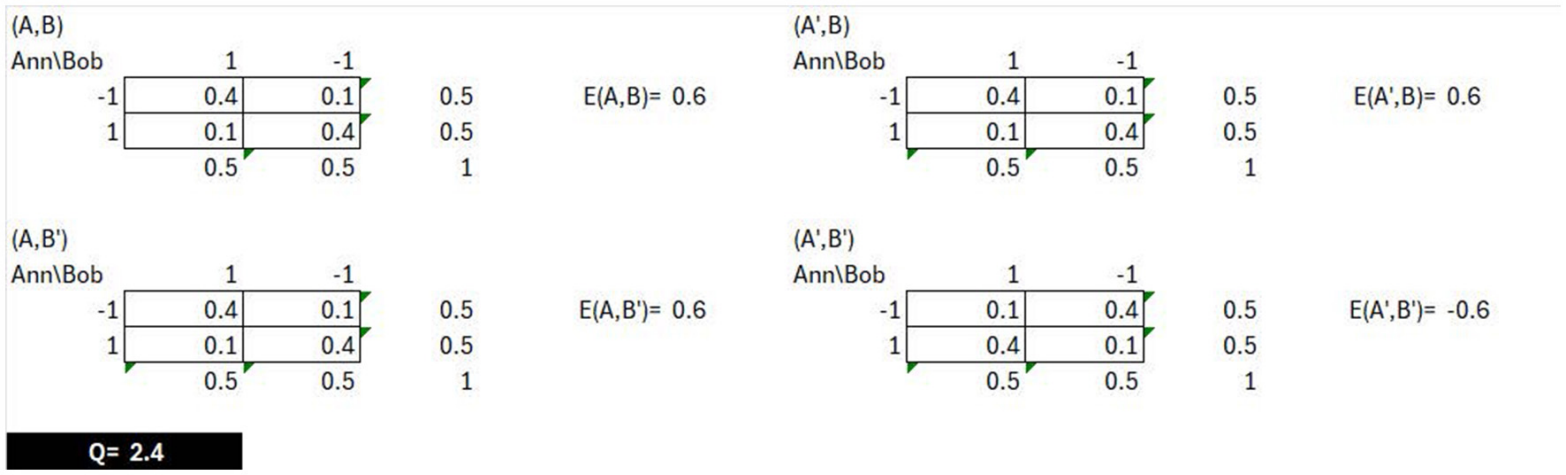
Local behavior, global correlations, and violation of CHSH inequalities.

However, the global structure of outcomes reveals a different pattern. Figure 1 represents the obtained signs, correlations, and Q.

Therefore, no assignment of fixed, context-independent properties to the product can reproduce these correlations. In other words, it is impossible to assume that the product possesses a predefined set of attributes determining all outcomes consistently across all interrogations.

This result exposes a fundamental tension. Locally, each observer performs identical and reproducible experiments, and observes stable statistical outcomes, leading to the natural conclusion that the product has intrinsic properties. Yet globally, the pattern of correlations violates classical constraints, showing that such an assumption cannot hold.

The resolution lies in recognizing that interrogative acts do not merely reveal properties but participate in the construction of outcomes. The correlations do not arise from a shared underlying cause, but from the global structure generated by the composition of local acts. Crucially, this structure remains inaccessible at the level of local observations: no observer can detect, from their own data alone, any deviation from classical behavior.

In this sense, the system exhibits quantum-like behavior. Local acts contribute to a non-classical global structure, while the no-signaling condition ensures that this contribution remains locally invisible. The apparent objectivity of the product is thus not fundamental but emerges from the stability of local statistics despite the presence of non-classical correlations.

If we consider *N* isolated subjects, Equation 3 can be transformed taking into account the Mermin's approach to many observers (Mermin, 1990) in the following way:


$$\begin{array}{l} Q\leq 2\sqrt{1+\left(2^{N-1}-1\right)\Delta^{2}} \end{array}$$ (5)

For *N* = 2, the bound reduces to $Q\leq 2\sqrt{1+\Delta^{2}}$, recovering the Tsirelson bound $2\sqrt{2}$, Δ = 1, and the classical CHSH limit when Δ = 0. For *N*≥3, the bound reproduces the exponential scaling of Mermin-type inequalities in the ideal case Δ = 1, while extending them by incorporating signaling (accessibility) and coherence effects through the parameter Δ.

Equation 5 is a global measure of multipartite correlations for an *N*-partite system where, as said, Δ = (1−*A*)(1−*C*) quantifies the effective activation of non-classical correlations through the interplay between global coherence *C* and accessibility *A*. Unlike standard Bell-type quantities, where $Q=|\langle M_{N}\rangle |$ represents the expectation value of the Mermin's operator, here *Q* is interpreted as the Euclidean norm of a vector in correlation space, combining classical and quantum contributions as $Q^{2}=Q_{classical}^{2}+Q_{quantum}^{2}$. The factor 2^*N*−1^−1 corresponds to the number of non-trivial global correlation channels, reflecting the discrete combinatorial structure of multipartite correlations, while Δ^2^ modulates their effective activation as a continuous parameter. In this framework, the classical limit (*A* = 1, *C* = 1) yields *Q* = 2, whereas the maximally non-classical regime (*A* = 0, *C* = 0) saturates the Mermin bound *Q* = 2^(*N*+1)/2^, providing a continuous interpolation between classical and quantum behavior. This construction therefore shifts the interpretation of *Q* from a single observable to a geometric measure of the total correlation structure, where the discrete dimensionality of the correlation space and the continuous degree of coherence jointly determine the magnitude of multipartite non-classicality. Although both constructions originate from the same set of multipartite correlators, Equation 5 and Mermin's approach differ fundamentally in how these contributions are combined. In the standard framework, the quantity $Q=|\langle M_{N}\rangle |$ is a *linear combination* of 2^*N*−1^correlation terms with alternating signs. This structure allows for constructive and destructive interference, so that *Q* effectively measures the projection of the correlation structure along a particular direction in correlation space. By contrast, in the present model the quantity $Q\leq 2\sqrt{1+\left(2^{N-1}-1\right)\Delta^{2}}\;$arises from *a quadratic combination* **o**f correlators, corresponding to a Euclidean norm. In this case, the individual contributions do not interfere destructively but are summed in quadrature, yielding a global measure of the total correlation strength. Thus, while the Mermin operator probes a specific observable associated with non-locality, the present formulation reinterprets *Q* as the magnitude of a vector in correlation space, providing a geometric characterization of the overall correlation structure rather than a directional measurement. In this approach *Q* characterizes the total non-classical correlation strength rather than a specific Bell-type violation.

### 6.2 A single agent

When a single agent applies a unique criterion, i.e., self-produced signs, to alternatives, this scenario generates only classical correlations.

When a single agent applies two distinct interrogative acts to the same object, the resulting outcomes may depend on the order and context of evaluation. This reveals an incompatibility between acts, as responses cannot be reduced to a single set of pre-existing properties. While this does not yet produce non-classical correlations of the Bell type, it already indicates that the object cannot be described independently of the interrogation procedure.

If, in addition, the evaluation processes share operational resources (such as time, memory, or control by a single agent), then a form of signaling between contexts can arise. In this case, the outcome in one context may depend on the choice or implementation of another, not through non-local interaction, but through shared internal mechanisms. However, in this regime, the contexts are no longer independent: they become correlated through the shared allocation of resources. This correlation reflects a coupling induced by operational constraints, rather than an intrinsic property of the object or a fundamental interaction between distinct systems.

An especially relevant case of signaling arises when time is treated as a scarce resource. If a single agent operates under finite temporal constraints, different interrogative acts cannot be implemented independently, as they compete for the same temporal budget. In such conditions, the choice to perform one act necessarily affects the execution of others, introducing a dependency between contexts.

This generates a form of signaling between contexts: the outcome in one context may depend on how time is allocated to other contexts. Importantly, this signaling does not arise from non-local interactions, but from the sequential and resource-limited nature of decision-making. It reflects an operational constraint rather than a fundamental violation of no-signaling. We call this condition *operational signaling*. In case of operational signaling *A* = *1*, C < 1 so that the thresholds for *Q* remain classical.

However, quantum-like behavior can emerge from the following scenario. Consider a single agent evaluating the same product through two different interrogative acts, A and A′, corresponding, for instance, to assessing quality and innovation. The agent performs these evaluations sequentially under a crucial constraint: after each evaluation, the outcome is not retained. That is, the agent has no memory of previous responses when performing a new interrogation. Two experimental sequences can be considered. In the first, the agent applies A followed by A′. In the second, the agent applies A′ followed by A. Suppose that empirical observations show that the correlation between outcomes depends on the order of evaluation: when A is followed by A′, the outcomes are positively correlated [e.g., E(A, A′) = 0.6], whereas when A′ is followed by A, the outcomes are negatively correlated [e.g., E(A′, A) = −0.6]. This indicates a non-commutative structure, as the order of interrogations affects the joint statistics. A crucial distinction must be drawn between single-subject and multi-subject scenarios. In the first case, a single agent evaluates multiple signs, and both acts and their compositional structure are, in principle, accessible. Non-classicality arises from the non-commutativity of interrogative acts and the impossibility of embedding local contexts into a global one. In a multi-subject scenario, different agents evaluate the same sign, which itself is the result of prior acts that remain inaccessible. Here, non-classicality is reinforced by the opacity of the sign's origin. The shared object does not function as a neutral input, but as a compressed trace of operations that cannot be reconstructed by any individual agent.

As a result, correlations between agents do not arise from jointly accessible operations, but from the interaction between local acts and globally produced, yet opaque, signs. This introduces a deeper form of non-separability, not only between acts, but between agents and the generative structure of the observed signs.

This is reminiscent of the Condorcet paradox (Arrow, 2012), where choices can be locally coherent but globally inconsistent: each pairwise comparison makes sense on its own, yet all comparisons together cannot be combined into a single consistent ordering. In this way, the system resembles quantum-like situations in which local consistency does not guarantee the existence of a coherent global description.

## 7 Advantages of a non-preference centric approach

In the standard quantum framework, entangled states are taken as fundamental global objects from which local probabilities are derived. In the present approach, this perspective is reversed: local measurement statistics are primary, and the global structure is defined only implicitly through their compatibility relations.

Entanglement is thus reinterpreted not as a property of a global state, but as a manifestation of the impossibility of constructing a fully consistent global configuration from locally observed probabilities. The parameter Δ = (1 – A)(1 – C) quantifies the portion of global inconsistency that remains inaccessible locally and determines the extent to which correlations deviate from classical bounds.

The introduction of self-produced stimuli does not require the assumption that all alternatives are simultaneously mentally represented or entangled prior to choice. Instead, alternatives may be sequentially constructed or selectively attended through internally generated cues or signs. For a choice to occur, one self-produced stimulus is adopted, guiding and stabilizing the selection of a specific alternative. On this view, choice is produced by the adoption of self-generated stimuli, which generate contextual correlations during the decision process through the selected sign, rather than by resolving a pre-existing superposed state. Consequently, the correlations between these stimuli emerge from the context in which they are applied, highlighting the constructive and context-dependent nature of decision-making.

Self-produced signs may be mutually incompatible and serve as detectors of fundamental properties of choice alternatives. These properties, together with the contextual structure of the decision process, generate correlations that can violate CHSH inequality.

Even if self-produced signs are partially guided by features of the choice alternatives, the resulting correlations cannot be reduced to these features. Rather, they reflect the context-dependent and constructive process of applying the signs to make a choice.

This approach can be adopted for analyzing and modeling decision-making of complex choices where self-produced stimuli (e.g., heuristics) force choice. The approach is also useful to model experimental conditions in which the types of alternatives were not known before experimentation.

### 7.1 Emergence, accessibility, and agent-based models

The relationship between local and global structures suggests an analogy with complex systems.

In standard agent-based models, global structure is typically understood as the emergent outcome of local interactions: agents follow simple rules, and collective patterns arise from their repeated interactions. Within this perspective, the global level is, at least in principle, reconstructible from local dynamics, implying both high global coherence and high accessibility. However, the present framework introduces a crucial refinement by explicitly distinguishing between the *emergence* of global structure and its *accessibility* from local observations.

While local interactions may indeed generate a form of global organization (*C*>0), this structure does not need to be fully accessible (*A* < 1). This leads to the definition of a residual quantity Δ = (1−*A*)(1−*C*) (see Equation 1), which captures the portion of global inconsistency that remains hidden from local observation. In this regime, the system exhibits quantum-like features: local behaviors are individually coherent and well-defined, yet they cannot be embedded into a single global classical description.

In classical agent-based models, by contrast, one typically assumes *A* = 1 and *C* = 1, allowing a full reconstruction and interpretation of the global state from local interactions. In practice, however, global structure in complex systems is often accessed only through statistical reconstruction. Standard statistical methods implicitly assume the existence of a single globally coherent probability distribution, thereby enforcing an effective global coherence even when the underlying system may not possess it. Consequently, hidden global inconsistency may be systematically masked, and genuinely non-classical features can be overlooked.

The key implication of this framework is that emergence alone is not sufficient to characterize complex systems. What becomes decisive is the interplay between emergence and accessibility. Global structure may arise from local interactions, but if it remains only partially accessible, part of its organization cannot be captured within a classical probabilistic model. It is precisely this partial inaccessibility that gives rise to genuinely quantum-like behavior, where correlations exist without being reducible to a single classical global description. The key point is that this global inconsistency is not due to lack of knowledge but to the structure of the dynamics itself: there is no single global state that integrates all possible orders of interaction. This resembles a key feature of quantum systems, where non-commuting operations prevent a complete, context-independent description. Thus, a globally non-classical structure can emerge from purely classical local behavior, remaining invisible to the agents who produce it.

In a quantum Condorcet framework, local pairwise preferences are individually consistent but cannot be extended to any coherent global ranking, reflecting an underlying contextual structure. Once the election outcome becomes accessible, this structure collapses, and the system reduces to a classical emergent inconsistency, since agents can now observe the contradiction they collectively generated.

It has not escaped our attention that, in an interconnected world, it is extremely difficult to observe quantum effects.

### 7.2 The role of researcher

On the one hand, a researcher generally observes only choices among alternatives, not the underlying experiments that subjects perform and that determine their decisions. The probabilities of choice can be contextual, arising from the experiments themselves and from the properties of the objects involved in the selected experiments. The researcher can only register correlations among the dimensions used as proxies for hidden signs. If these correlations are non-classical, then no classical hidden-variable distribution over the adopted measures can explain them. In other words, it is as if the decision outcome depends on the context created by the subjects' own experiments and on the properties of the objects involved, rather than being predetermined by a prior cause.

On the other hand, the second role of the researcher is to design experiments to test assumptions involving decision-makers. In this case, human beings are forced to follow prescribed experiments in order to make a choice. This, however, does not guarantee that self-produced signs are not produced. Again, observing the correlations among the imposed experiments and explicating the self-produced signs can reveal whether and which type of correlations emerge during and because of the experiments.

## 8 Conclusions

We presented a theoretical framework in which choices are mediated by self-produced signs, which may be mutually incompatible and function as detectors of fundamental properties of choice alternatives. These signs are generated endogenously in service of making a decision, and the correlations they produce are context-dependent, arising from the manner in which the signs are applied rather than from prior causes. While one might argue that object features influence the generation of signs, the resulting correlations, including CHSH violations, cannot be reduced to these features alone. Instead, they reflect the constructive, goal-directed, and context-sensitive dynamics of cognitive processing, highlighting that decision-making is an active and contextually emergent process rather than a simple unfolding of pre-existing causes. Importantly, this framework also avoids the need to posit overly forced superpositions of preferences on alternatives in the mind, offering a more flexible and realistic account of cognitive choice.

The research program introduced by this approach aims to identify which heuristics and types of self-produced signs are involved in choice, and how they generate contextuality that requires non-classical approaches to be analyzed.

In fact, for classical and rational decision-making theory, human beings choose alternatives by computing the utility they expect to receive and the probabilities that the consequences of different choices will occur (expected utility). Alternative models attempt to explain biases, bounded rationality, and violations of Bayes' rule by adopting modified formulations of expected utility in which probabilities may be subjective rather than objective, or context-dependent, but they still satisfy the axioms of classical (Kolmogorovian) probability theory.

By contrast, in QDT probabilities are not merely reweighted or distorted versions of classical probabilities. They are derived from a fundamentally different mathematical structure. Cognitive states are represented as vectors in a Hilbert space, and the probability of a choice is obtained through the quantum probability rule (Born rule). This implies that the order of questions or information can change the resulting probabilities before a decision, cognitive states can coexist in a structured indeterminate state rather than being reducible to a classical mixture (superposition); the probability of a choice may include constructive or destructive interference terms, which naturally account for violations such as the disjunction effect or conjunction fallacy.

Thus, quantum probabilities in QDM do not simply adjust classical expected utility; they replace the underlying probabilistic framework. The difference is not only quantitative (different probability values) but structural: uncertainty is modeled as an intrinsic feature of cognitive state representation, rather than as incomplete information about an objectively defined event space.

A central methodological concern regarding Quantum Decision Theory (QDT) relates to the epistemic status of the cognitive states it postulates. In QDT, decision makers are represented as occupying superposed cognitive states in a Hilbert space and observed choice probabilities are derived via the Born rule. However, these mental states are not directly observable; they are inferred from behavioral data. The structure of the Hilbert space, the choice of basis states, and the presence of interference terms are typically constructed to reproduce empirically observed deviations from classical predictions. This raises a critical question: does QDT genuinely predict behavior ex ante, or does it reconstruct it ex post using a mathematically flexible framework?

The concern is not trivial. If the relevant cognitive observables are defined by the experimental design itself, then the representation risks becoming model-dependent rather than theoretically grounded. In such cases, “superposition” may function as a descriptive device rather than as an independently motivated psychological mechanism. The danger is one of structural overfitting: with sufficient dimensional freedom, a Hilbert space model may accommodate a wide range of empirical patterns without providing strong constraints (Pothos and Busemeyer, 2013).

Defenders of QDT respond that quantum probability theory imposes non-classical but nonetheless strict algebraic constraints, such as non-commutativity and bounded interference effects, which limit arbitrary parametrization. Moreover, in domains such as question order effects, the framework yields specific, testable relationships among probabilities that go beyond *post hoc* curve fitting.

Still, the philosophical issue remains: is QDT an ontological account of operational architecture, or a powerful instrumental formalism for organizing systematic violations of Expected Utility Theory? The answer is the second if we adopt the current QDT approach (Holtfort and Horsch, 2023). It is the first one if we assess quantum correlations on outcomes of experiments human beings adopt to make choice.

### 8.1 Limitations

This study is primarily theoretical. However, to support the empirical relevance of the proposed framework, in Appendix A we outline a possible experimental protocol designed to test its main predictions.
