# Supplementary material for: Self-produced signs in decision-making and contextual correlations
Source: Front Psychol. 2026 Jul 9;17:1838592. doi: 10.3389/fpsyg.2026.1838592 (PMC13392539; doi:10.3389/fpsyg.2026.1838592)
Supplement: Supplementary file 1 [file Supplementary_file_1.docx]

**Experimental Protocol for Contextual Condorcet Structures**

*A.1. Objective*

The aim of the experiment is to test whether locally constructed decisions among alternatives can give rise to globally incompatible structures that cannot be reduced to a single underlying preference ordering. In particular, the protocol investigates whether pairwise choices among three alternatives (A, B, C), when generated through context-dependent internal processes (signs), produce correlations that do not admit a global joint representation.

*A.2. Theoretical Premise*

The proposed framework assumes that decision-making does not rely on stable, pre-existing preferences, but is actively constructed through the generation of self-stimuli (or “signs”) by the decision-maker. These internally generated elements guide decisions within each context. Individual choices are locally coherent; however, the global structure of decisions may fail to admit a consistent joint representation. This contrasts with classical models (including the standard Condorcet mechanism), where inconsistencies arise from aggregation of stable individual preferences, and with quantum decision models that introduce contextuality at a formal level without specifying an operational cognitive mechanism. The crucial difference with the classical Condorcet mechanism lies in the existence of a global representation: in the classical case, cycles arise from the aggregation of consistent individual preferences, which admit a joint probabilistic model; in the present framework, no such global model exists, as the observed correlations cannot be extended to a joint representation.

*A.3. Phase 1*

*A.3.1. Experimental Setup*

Participants are presented with three alternatives (A, B, C). The alternatives should be designed so as to avoid pre-existing familiarity, introducing novel options that discourage the use of established preferences and instead promote the construction of context-dependent decision criteria.

They perform pairwise comparisons: A vs B, B vs C, C vs A. Each comparison defines a distinct experimental context.

Two implementations are possible:

(i) Between-subject design: participants are randomly assigned to a single comparison context.

(ii) Within-subject design: participants perform multiple pairwise comparisons in randomized order but are not allowed to revisit previous responses.

*A.3.2. Context Isolation*

To preserve the contextual structure only one comparison is presented at a time. No feedback about previous or aggregate results is provided; participants cannot access their full set of responses during the task; memory effects are controlled (e.g., time constraints or distractor tasks). This ensures that each decision is made locally, without access to a global preference structure.

*A.3.3. Elicitation of Decision Processes (Signs)*

After each decision, participants are explicitly asked: “How did you arrive at this choice?” They are required to report the criteria, cues, or internal considerations guiding their decision. This can be implemented through short written responses; think-aloud protocols; selection among predefined categories. These responses operationalize the notion of “signs”, i.e., internally generated elements that mediate the decision process. Importantly, these signs are not interpreted as stable preferences, but as context-dependent constructs emerging during each decision.

*A.3.4. Data Collection*

Each outcome is encoded as: X = outcome of (A vs B), Y = outcome of (B vs C) Z = outcome of (C vs A) with values -1, 1.

For each trial, we record outcome (choice); response time (optional); reported decision process (signs). From these data, we will estimate marginal distributions P(X), P(Y), P(Z); pairwise correlations E[XY], E[YZ], E[ZX]; distributions of reported decision criteria.

*A.3.5. Structural Analysis*

Two complementary analyses are performed: (1) Transitivity and Condorcet Test. We evaluate whether outcomes are compatible with a transitive ranking. The presence of cycles (A > B, B > C, C > A) indicates global inconsistency. (2) Joint Representability (Contextuality Test). We assess whether a joint distribution P(X, Y, Z) exists that reproduces all observed pairwise statistics. Failure to construct such a distribution indicates contextuality, i.e., the impossibility of a global assignment despite local consistency. Optionally, a scalar quantity as defined in the main text (4) can be computed to quantify the degree of global inconsistency, dependence on the number of observers, level of local accessibility.

*A.3.6. Distinguishing from the Classical Condorcet Mechanism*

The proposed framework is empirically distinguishable from the classical Condorcet mechanism not at the level of aggregate outcomes, but at the level of decision construction. To test this, the protocol includes additional checks:

(a) Reconstruction of preferences: participants are asked to provide a global ranking after the task. Difficulty or inconsistency indicates absence of an underlying preference structure.

(b) Stability of decision criteria: reported signs are compared across contexts. Context-dependent variation indicates non-reducibility to a single preference ordering.

(c) Cross-context prediction:

Participants are asked to infer one comparison from others. Failure of consistency indicates lack of global structure. These tests allow distinguishing between aggregation of stable preferences (classical Condorcet) context-dependent construction of decisions (present framework).

*A.3.7. Role of multiple observers and isolation*

A sufficiently large number of participants is required to ensure statistical robustness and to amplify the detection of global inconsistency. Increasing the number of observers improves estimation of correlations and strengthens detection of incompatible structures. However, this amplification is meaningful only under strict isolation conditions: participants do not interact; no communication occurs between trials; no aggregate outcomes are disclosed; experimental conditions prevent shared contextual dependencies. This isolation ensures that observed inconsistencies cannot be attributed to signaling or shared resources but arise from independent local decision processes.

*A.3.8. Interpretation*

If data admit a global representation, results are consistent with classical models of preferences or bounded rationality. If data are locally consistent but globally incompatible, this supports a contextual interpretation. Importantly, participants do not observe global inconsistency directly. It emerges only at the level of aggregate analysis.

*A.3.9 Comparison with Alternative Models*

The protocol allows comparison between classical preference-based models; bounded rationality models (noise-driven inconsistencies); quantum-inspired decision models; the present framework, where contextuality arises from self-generated decision processes.

*A.3.10. Limitations*

The protocol focuses on minimal structures and controlled environments. Potential limitations include residual memory or learning effects; implicit shared constraints; simplification of real decision processes. Future extensions may include: more alternatives (n > 3); dynamic decision processes; explicit modeling of sign-generation; longitudinal studies of decision construction.

A potential concern is that, according to the principle of sufficient reason, participants will always provide some justification for their choices. Our framework fully accommodates this: each decision is locally supported by a reason. However, these reasons are context-dependent and need not be mutually consistent. The key prediction is therefore not the absence of justification, but the impossibility of integrating such local justifications into a single coherent global explanation.

*A.4. Phase 2.*

The second phase tests whether contextual structures persist or collapse once global information becomes accessible.

To investigate the role of global information in shaping decision processes, a second experimental phase is introduced following the initial data collection. The aim of this phase is to assess how access to aggregate or global information affects locally constructed decisions and the resulting contextual structure. After completing the initial pairwise comparisons (Phase 1), a subset of participants is exposed to additional information about the experiment. This may include aggregate outcomes (e.g., majority choices across participants; summaries of pairwise preferences.)

To control the experiment, access to global information is provided without allowing communication among participants, ensuring that any observed changes in behavior are attributable to informational effects rather than coordination or signaling.
